# Lung Function Abnormalities in Sickle Cell Anaemia

**DOI:** 10.1155/2019/1783240

**Published:** 2019-04-01

**Authors:** Yvonne A. Dei-Adomakoh, Jane S. Afriyie-Mensah, Audrey Forson, Martin Adadey, Thomas A. Ndanu, Joseph K. Acquaye

**Affiliations:** ^1^Department of Haematology, School of Biomedical and Allied Health Sciences, College of Health Sciences, University of Ghana, Accra, Ghana; ^2^Department of Medicine and therapeutics, School of Medicine and Dentistry, College of Health Sciences, University of Ghana, Accra, Ghana; ^3^Department of Community and Preventive Dentistry, School of Medicine and Dentistry, College of Health Sciences, University of Ghana, Accra, Ghana

## Abstract

**Background:**

Abnormalities in lung function tests have been shown to commonly occur in a majority of patients with sickle cell disease (SCD) even at steady state. The prevalence and pattern of these lung function abnormalities have been described in other populations but this is unknown among our sickle cell cohort. There is generally little information available on risk factors associated with the lung function abnormalities and its relevance in patient care.

**Method:**

This was an analytical cross-sectional study involving 76 clinically stable, hydroxyurea-naive adult Hb-SS participants and 76 nonsickle cell disease (non-SCD) controls. A structured questionnaire was used to obtain sociodemographic data and clinical history of the participants. Investigations performed included spirometry, pulse oximetry, tricuspid regurgitant jet velocity (TRV) measurements via echocardiogram, complete blood counts, free plasma haemoglobin, serum urea, and creatinine.

**Results:**

Weight, BMI, mean FVC, and FEV1% predicted values were comparatively lower among the Hb-SS patients (p < 0.001). Abnormal spirometry outcome occurred in 70.4% of Hb-SS patients, predominantly restrictive defects (p < 0.001), and showed no significant association with steady-state Hb, WBC count, free plasma haemoglobin, frequency of sickling crisis, chronic leg ulcers, and TRV measurements (p > 0.05). The mean oxygen saturation was comparatively lower among Hb-SS patients (p < 0.001).

**Conclusion:**

Measured lung volumes were significantly lower in Hb-SS patients when compared to non-SCD controls and this difference was not influenced by anthropometric variance. Lung function abnormalities, particularly restrictive defects, are prevalent in Hb-SS patients but showed no significant association with recognized markers of disease severity.

## 1. Introduction

The lung is one of the major organs affected by sickle cell disease (SCD) being a common site of hypoxic and ischaemic injury and emboli from marrow infarcts/fat necrosis and is plagued with increased propensity for developing pneumonias [1–3]. Acute and chronic lung complications in sickle cell disease are major causes of morbidity and mortality, accounting for 20-30% of all sickle cell deaths [1, 4]. Chronic complications could manifest as abnormalities in lung function, interstitial lung disease, or pulmonary hypertension. Lung function abnormalities have been shown to commonly occur in a majority of SCD patients even at steady state and these include lower lung volumes (FEV1/FVC), decreased diffusion capacity for carbon monoxide, airway hyperresponsiveness/asthma, and hypoxaemia [5, 6]. The reported prevalence of these lung function abnormalities is varied [7–11]. Although early stages of these lung function abnormalities are usually asymptomatic, a significant number of SCD patients may have dyspnoea on exertion with reduced exercise capacity when severe [12]. A recent study found an association between low FEV1 and increased risk of mortality among adults with Hb-SS [13]. Powars et al. had earlier suggested that abnormalities in lung function tests could be the first objective sign of sickle cell chronic lung disease and could reduce morbidity when detected early through screening [10]. The current study therefore sought to determine the prevalence and pattern of lung function abnormalities among a cohort of adult Hb-SS patients compared to healthy non-SCD controls and identify associated factors.

## 2. Methods

This was an analytical cross-sectional study carried out between March and June 2014. The study was conducted at the Ghana Institute of Clinical Genetics which houses the sickle cell clinic of the Korle-Bu Teaching Hospital, a major referral site for the Accra Metropolis. The study sample consisted of 76 randomly selected Hb-SS adults, 18 years and above from the out-patient unit of the sickle cell clinic who were in their steady state with results of electrophoresis clearly documented in their medical records. Seventy-six-year-old and sex matched non-SCD controls were selected from among the healthcare staff of the above hospital.

A structured questionnaire was used to collect participants' sociodemographic and medical information after written consent was obtained. Anthropometric measurements such as weight, height, and BMI were taken as well as blood pressure, pulse rate, and oxygen saturation using Omron digital BP machine (Intelli Sense R) and ChoiceMMED brand pulse oximeter, respectively. A modified version of St. George's Respiratory questionnaire was used to screen the participants for symptoms of chronic respiratory diseases and excluded if present. General cardiorespiratory examination was performed on all participants to further exclude respiratory, cardiovascular, and musculoskeletal abnormalities that were capable of influencing the spirometry or echocardiogram results. Spirometry was performed and interpreted for all participants according to American Thoracic Society (ATS) protocol using SCHILLER SPIROVIT SP-1 (Schiller-AG, Switzerland) [14]. The best of three FEV1 and FVC test value from acceptable and reproducible maneuvers was recorded as both absolute and percentage predicted values. Abnormal FEV1 or FVC% predicted was defined as values < 80% of predicted values. For the purposes of this research, a forceful blow of ≥ 3sec was considered an acceptable blow for interpretation and analysis [9]. Blood samples taken were analysed for complete blood count, urea, and creatinine and free plasma haemoglobin (a marker of intravascular hemolysis). For the latter, solid phase-phase sandwich ELISA technique was used and optimization was predetermined prior to running the test sample. Values >40ug/ml were regarded as abnormal and suggestive of ongoing intravascular haemolysis [3]. Patients underwent transthoracic echocardiogram which was performed by a consultant cardiologist using a Toshiba brand ECHO machine, Aplio 300 3MHz transducer in accordance with the American echocardiography society guidelines [15]. The average of three different TRV measurements was recorded as the mean TRV value, where values ≥ 2.5m/s were classified as elevated TRV [16].

### 2.1. Study Definitions

Steady state: absence of acute painful events or hemolytic episodes 4 weeks prior to recruitment as well as no hemotransfusion within the same specified period.

Sickle cell crisis: sickle cell crisis was defined as acute painful episodes that required use of oral or intravenous analgesics and led to ER or daycare visits with or without hospitalization.

Severity of disease: for the purpose of this study disease severity was defined as (1) having 3 or more acute painful episodes in the past one year prior to study [17]; (2) history of chronic leg ulcer (3) history of priapism; (4) elevated TRV on ECHO.

Data analysis was done using Statistical Package for Social Sciences (SPSS) version 22. ANOVA was used to compare FVC and FEV1% predicted values among the study participants as well as comparing means of laboratory indices between Hb-SS participants with normal and abnormal spirometry outcomes. Spearman's rank correlation and Dunnett's pairwise post hoc analysis were used to establish relationships between spirometry and laboratory measurements. Chi-square test was employed to compare proportions of spirometry outcomes among relevant categories of respondents. Linear regression and multivariate analysis were used to examine significant factors influencing spirometry outcomes.

Approval was obtained from the Ethical and Protocol Review Committee of the College of Health sciences, University of Ghana (protocol identification number CHS-Et/M.10-P3.9/2013-2014).

## 3. Results

Table 1 shows the age and gender distribution as well as anthropometric characteristics of the participants. The mean age for the Hb-SS participants was 33.8 ± 11.1 years with majority (60.5%) being females. The mean weight and BMI of Hb-SS participants were 59±13.2kg and 21.8±4.2, respectively, compared to 69.1± 14.5kg and 25.1± 4.8, respectively, among the controls (p < 0.001). Both groups had comparable mean height of 164. 3±cm and 166.1 ± cm, respectively (p = 0.077).

### 3.1. Clinical History of the Hb-SS Subjects

Out of the 76 participants, 6 (7.9%) had associated hypertension, only 1 (1.3%) had diabetes, and 1(1.3%) had chronic kidney disease (CKD). Fifty-three (69.7%) had a history of previous blood transfusion and 28 (36.8%) had a history of chronic (present/past/recurrent) leg ulcers. Nine (25.7%) out of the 30 males had a previous history of priapism, mostly in the adolescent period. Sixty-three (82.9%) had at most 2 episodes of vasoocclusive (VOC) crisis in the year prior to the study, while 13 (17.1%) had 3 or more episodes over the past year.

### 3.2. Lung Function Parameters of Hb-SS Compared to Non-SCD Controls

Five Hb-SS participants and 2 of the controls had poor spirometry tests (forceful blow duration less than 3secs); thus their results were not included in the analysis. From Table 1, the mean FVC and FEV1% predicted of the Hb-SS patients were 72.6 ± 22.2% and 65.3 ± 20.6%, respectively, compared to 90.4±11.9% and 87±12.8% among the controls (p < 0.001). Analysis showed that 53 (74.6%) and 43 (60.6%) of the Hb-SS participants had abnormal FEV1% and FVC% predicted, respectively (<80%).

Table 2 showed that FEV1 and FVC% predicted values positively correlated with the age, height, weight, and BMI of the participants but these were not statistically significant except for weight and BMI (p<0.05). Gender was also significantly associated with participants FEV1% predicted (p=0.010).

Logistic regression analysis however showed that age, height, gender, weight, and BMI did not significantly influence the presence or absence of abnormal FEV1 and FVC% predicted (Table 3). Also, the odds of having abnormal FEV% and FVC% predicted among the cases were 11.7 and 13.2, respectively (Table 3).

As shown in Table 4, 50(70.4%) of Hb-SS patients compared to 9(12.2%) of the controls had abnormal spirometry outcome (defined as restrictive, obstructive, or mixed restrictive and obstructive defects; p < 0.001). The proportions of abnormal spirometry outcomes among the Hb-SS participants were restrictive (70%), obstructive (16%), and mixed defects (14%), while the controls all had restrictive defects only. The mean oxygen saturation was 94.0±1.9% on room air compared to 97±1.6% among the controls (p < 0.001). There was no significant difference between the mean oxygen saturation of Hb-SS participants with normal or abnormal spirometry outcome.

### 3.3. Association between Laboratory Parameters and Spirometry Outcome of Hb-SS Participants

The mean steady-state haemoglobin of the Hb-SS participants was 8.1 ± 1.6g/dl with a range of 4.90 – 11.2 g/dl. The mean total WBC was elevated (10.1 ± 3.6 x 10^9^/l) with a range of 3.4–26 x10^9^/l. The mean platelet count was normal 341.4 ± 109.2 x10^9^/l showing a wide range of 143-873 x 10^9^/l. Mean blood urea was normal, 3.5 ± 1.6 mmols/l with a mean creatinine of 55.6 ± 20.0 mmols/l. Mean free plasma haemoglobin was elevated, 105.7 ± 53.4*µ*g/ml with 66 (91.7%) of the patients having values > 40 *µ*g/ml, indicative of intravascular haemolysis.

From analysis, abnormal spirometry outcome was significantly associated with blood urea level (p=033) but not with serum creatinine (p=0.056). A positive correlation was observed between FEV1% predicted and haemoglobin level; R=0.239; p = 0.043 (Table 5). We also found that free plasma Hb levels > 40*µ*g/ml was significantly associated with FEV1% (p= 0.031). However when other laboratory factors were accounted for using linear multivariate regression analysis, none of the laboratory factors including Hb, urea, and free plasma Hb were significantly associated with FEV1% predicted. Comparing laboratory parameters among the Hb-SS participants with various spirometry defects, total platelet count appeared to be significantly lower in those with obstructive defects; p=0.034 (Table 6). Further post hoc analysis for pairwise comparison using Dunnett's test showed that only obstructive and restrictive defects were nearly significantly different (p=0.05), suggesting a lower platelet count among patients with obstructive defects.

Also, TRV, a noninvasive marker of elevated pulmonary artery systolic pressure showed a tendency to be comparatively elevated in those with mixed restrictive and obstructive defect (p=0.056). However, there was no significant association between TRV and percentage predicted values of FEV1 and FVC (Table 7).

### 3.4. Spirometry Measurements and Associated Clinical Factors

Hb-SS participants with ≥ 3 sickle cell crisis in the past year had a lower mean FVC% predicted (65.9%) compared to a mean of 75.0% in those with ≤ 2 crisis in the past year (p=0.180). There were no significant differences between participants with or without history of chronic leg ulcer with regard to their FVC and FEV1% predicted values.

## 4. Discussion

### 4.1. Participants' Demographic Characteristics

Generally, children as well as adults with sickle cell disease are believed to be of a lower average body weight and height than their unaffected peers [18–20]. A study by Oguntoye et al. in Nigeria reported that, until the age of 18, the trend in height and weight of children with sickle cell disease is identical to that of their unaffected peers but began to show significant differences after 18 years [21]. They also showed that the gap in anthropometric measurements between the two groups further widened with increasing age. On the other hand, some studies have found no significant height variation among SCD patients 18 years and above when compared to healthy controls, in fact reporting average or above average heights [22, 23]. The current study similarly showed that the Hb-SS subjects were comparable in height to the controls but had significantly lower body weight and BMI. The negative effect of sickle cell disease on growth and development, especially during the adolescence period, has been blamed for this variance; however, the role of nutrition cannot be overlooked [24]. Incidentally the average BMI of our Hb-SS participants (21.30) was comparable to that of the Hb-SS participants (21.80) in a US study by Klings et al. [9]. This could imply comparable nutritional status of the Ghanaian Hb-SS patients to that of the American cohort but improved holistic medical care of sickle cell patients in our part of the world, especially during early childhood, could also influence this pattern.

### 4.2. Spirometry Parameters and Anthropometry of Participants

The current study showed that measured lung volumes (FEV1 and FVC% predicted) were significantly lower in Hb-SS patients compared to the non-SCD controls and this is consistent with findings from previous studies [6, 22, 25]. Age, gender, standing height, and weight are important determinants of lung function due to their significant correlation with Pulmonary Function Tests and this correlation has similarly been shown in our study [26]. With the established disparity in anthropometric parameters particularly weight, BMI, and sometimes height, it is believed that the observed lower lung volumes in SCD patients is largely a result of this disparity and not necessarily pathological [27]. In addition, Miller and Sergeant proposed that, aside from the anthropometric variance, SCD patients have a relatively small chest wall compared to their body size (disproportionate chest wall growth) which is believed to partly contribute to the observed decrease in lung volumes [27]. The suggested reason for the disproportionate chest wall growth was repeated infarctions in the ribs, sternum, and vertebra that impair optimal skeletal growth and development.

In the current study, however, multiple regression analysis showed that the significant variance in weight and BMI could not explain the observed wide differences in lung function measurements between the Hb-SS participants and the controls. Also the odds of having an abnormal FEV_1_ and FVC% predicted in Hb-SS patients were about 12 and 13 times, respectively, the risk in the healthy group meaning the presence of the disease predisposes to abnormal lung function. These suggest that reduced lung volumes in Hb-SS patients comparative to non-SCD controls may result from a combination of factors/complications of the disease and not merely due to anthropometric variance although the latter may be contributing to it. It can be inferred from this analysis that the mere presence of Hb-SS is a significant predictor of abnormal lung function. Some studies have reported diminished respiratory muscle strength in children and young adults with SCD which could also partly explain the lower lung volumes [28, 29].

### 4.3. Prevalence of Abnormal Spirometry among Hb-SS Patients and Associated Factors

The study found that a significant proportion (70.4%) of the Hb-SS participants had abnormal spirometry outcome, with restrictive defect being predominant. In a US study by Klings et al., as much as 90% of the Hb-SS participants had abnormal lung function including 13% with impaired DLco [9]. Our proportion was comparatively lower and this could be due to our inability to test for diffusion capacity (DLco). Similar to our study, Dosunmu and colleagues in Nigeria found that 68% of the SCD participants had abnormal spirometry outcome, of which 53% had restrictive defects, 3.7% obstructive defects and 11% with mixed defects [8]. These consistent results more prove the predominance of restrictive defects among adults with sickle cell disease [6–9, 30, 31]. Abnormal lung function, particularly, restrictive defects in Hb-SS, has been shown to be more prevalent in those with a severe disease pattern evident by recurrent vasoocclusive crisis and ACS, lower haemoglobin, raised markers of haemolysis, chronic leg ulcers, renal dysfunction, and raised pulmonary arterial pressures/pulmonary hypertension [5, 6, 16, 32].

A prospective study by Aquino and colleagues reported a significant correlation between the severity and extent of parenchymal abnormalities on chest CT-scan and the number of prior ACS [33]. Maioli and colleagues in Brazil also found an association between a history of ACS and restrictive defects on spirometry [34]. In our study, we were unable to obtain a definite history of ACS due to diagnostic challenges that existed but observed that patients with increased frequency of crisis (3 or more in the past year), largely VOC in nature, had lower FVC % predicted values when compared to those with 2 or less episodes of sickle cell crisis in a year (65.9% predicted versus 75.0% predicted, respectively; p=0.180). This supports the notion that recurrent VOC crisis could cause ischaemic damage to the lung parenchyma with fibrosis via the release of inflammatory mediators from the damaged endothelium and hypoxic lung and hence the decrease in lung volumes [35, 36]. Obstructive defects, however, have been reported to be more prevalent among children with SCD in whom asthma-like symptoms and airway hyperreactivity could occur [37, 38]. The study by Klings et al. [9] and Dosunmu et al. [8] found low prevalence of obstructive defects in the adult SCD population studied. In contrast, Vendramini and colleagues [25] however reported a high prevalence (31%) of obstruction and airway hyperreactivity among adults with SCD. Our proportion of adult SCA patients with obstructive defects was 16% which was similar to 19.5% observed by Williams et al. [6]. The causes of obstruction in SCD are more complex and quite unclear but thought to include inflammation, effects of hypoxia, and oxidative stress on the bronchial tree [25].

Sickle cell vasculopathy is a complication in SCD attributed to the toxic effects of products of intravascular haemolysis and has been linked to the development of pulmonary hypertension, chronic leg ulcers, chronic kidney disease, and chronic lung complications in sickle cell patients [39, 40]. Similar to the above observation, our analysis showed that free plasma Hb levels above 40ug/ml, evident of intravascular haemolysis, were significantly associated with abnormal FEV1% predicted (< 80% predicted) and this was consistent with findings by Sylvester et al. who found an association between FEV1% predicted and markers of haemolysis among SCD patients [41]. We also found that steady-state haemoglobin showed a positive correlation with FEV1% predicted but Williams and colleagues rather found an association between steady-state Hb and FVC % predicted; p < 0.001 [6, 42]. However on linear multivariate analysis, Hb or free plasma haemoglobin significantly predicted abnormal FEV1%.

Our study also showed a significant association between abnormal spirometry outcome and blood urea level (p = 0.033) with a rising trend of creatinine level in those with abnormal spirometry (p = 0.056). These observations support suggestions from previous studies of a possible common aetiological link between chronic lung disease and kidney disease in SCD, likely through sickle cell associated vasculopathy [9].

It has been proposed that activated white blood cells and platelets promote vascular inflammation and vessel damage. They are therefore likely to play a role in initiating sickle cell vasculopathy and hence the development of perivascular lung parenchymal fibrosis with effects on measured lung volumes [35]. Dosunmu and colleagues reported a negative correlation between total WBC and platelet counts with the FVC% predicted among the studied SCD cohort [8]. We observed that the mean platelet count had a tendency to be lower among those with obstructive defects (p =0.05). Studies by Gladwin et al. and Anthi et al. have shown that SCD patients with raised pulmonary artery systolic pressure usually show a restrictive pattern on spirometry [16, 43]. We have shown that TRV had a tendency to be higher in Hb-SS patients with spirometry defects, particularly in those with mixed defects (TRV of 2.6 ± 0.4; p = 0.056).

In conclusion, the current study has shown that lung volumes are significantly lower in Hb-SS patients when compared to non-SCD controls. The mere presence of the disease strongly predicts abnormal lung function. With the high prevalence of lung function abnormalities among Hb-SS patients, one may suggest that the former could itself be considered a marker of disease severity and hence periodic screening with spirometry may be necessary to allow for patient categorization and further management.

## Figures and Tables

**Table 1:** Demographic characteristics and lung function parameters of participants.

	N	Mean	Std. Deviation	Minimum	Maximum	Sig.
*Weight (kg)*						
Hb-SS subjects	76	59.00	13.20	38.00	117.50	<0.001
Controls	76	69.10	14.50	46.00	115.00	
*Height (cm)*						
Hb-SS subjects	76	164.30	8.20	135.00	182.50	0.077
Controls	76	166.10	8.24	150.00	190.00	
*BMI*						
Hb-SS subjects	76	21.80	4.20	15.80	40.20	<0.001
Controls	76	25.05	4.77	17.00	39.00	
*Age (years)*						
Hb-SS subjects	76	33.75	11.11	18.00	63.00	0.970
Controls	76	33.81	9.81	18.00	61.00	
*FVC *%* Predicted*						
Study subjects	71	72.63	22.19	40.00	120	0.001
Controls	74	90.38	11.85	53.00	114	
*FEV1 *%* Predicted*						
Study subjects	71	65.32	20.55	24.00	115.00	0.001
Controls	74	87.03	12.84	50.00	115.00	
%* Ratio (FEV1/FVC)*						
Study subjects	71	75.64	9.59	50.20	92.20	0.001
Controls	74	82.16	6.28	67.50	100.00	

**Table 2:** Relationship between FEV1, FVC percentage predicted values, and anthropometric variables of participants using Spearman's rank correlation analysis.

		Height (n=144)	Weight (n=142)	BMI (n=139)	Age (145)
FEV1 %Predicted	Spearman's rank Correlation	0.089	0.271	0.243	0.071
	Sig. (2-tailed)	0.287	**0.001**	**0.004**	0.397

FVC % Predicted	Spearman's rank Correlation	0.041	0.187	0.177	0.108
	Sig. (2-tailed)	70.627	**0.026**	**0.037**	0.197

**Table 3:** Factors that influence abnormal FEV1 and FVC% predicted categories.

FEV1% Category					FVC% Category			
	P-value	Exp(B)	95% CI		P-value	Exp(B)	95% CI	
		(OR)	Lower	Upper		(OR)	Lower	Upper
Weight	0.686	0.952	0.751	1.207	0.321	0.885	0.695	1.127
Height	0.982	1.002	0.826	1.216	0.282	1.113	0.915	1.354
BMI	0.626	1.175	0.614	2.247	0.245	1.475	0.766	2.841
Age	0.643	0.991	0.953	1.03	0.332	0.981	0.945	1.019
Gender(1)	0.211	0.573	0.239	1.372	0.202	0.564	0.234	1.361
Case-Control (1)	**<0.001**	**11.69**	4.844	28.246	**<0.001**	**13.153**	**5.139**	**33.662**
Constant	0.913	0.17			0.22	<0.001		

**Table 4:** Spirometry outcomes among participants.

Spirometry results categorization	Respondent Category		
	Study subjects	Controls	
	n (%)	n (%)	P-value
Normal	21 (29.60)	65 (87.80)	
Abnormal	50 (70.40)	9 (12.20)	<0.001
Total	71 (100.00)	74 (100.00)	

**Table 5:** Correlation between FEV1, FVC percentage predicted, and laboratory parameters using Spearman's ranked correlation coefficient Rho.

Laboratory indices	FEV1%		FVC%	
	Spearman's Coefficient Rho	P-value	Spearman's Coefficient Rho	P-value
Haemoglobin(g/dl)	0.244	**0.043**	0.200	0.100
Total WBC	0.081	0.514	0.058	0.637
Platelet	0.155,	0.208	0.010	0.938
Plasma Hb	- 0.108	0.387	- 0.115	0.359
Urea	- 0.067	0.568	- 0.113	0.393
Creatinine	- 0.039	0.774	0.032	0.810

**Table 6:** Comparisons of means of laboratory results and TRV with spirometry outcomes among Hb-SS subjects.

Spirometry Interpretation					
	Obstructive	Restrictive	Mixed	Normal	P-value
Hemoglobin Level (g/dl)	8.4±1.8	7.9±1.6	7.3±2.0	8.4±1.3	0.350
Total Neutrophil count (x 10^9^/l)	8.5±3.2	10.6±4.0	8.3±1.2	10.3±3.3	0.413
Platelet Count (x 10^9^/l)	251.1±58.9	366.6±103.1	308.4±87.8	350.6±127...3	**0.034**
Plasma Haemoglobin (ug/ml)	94.8±46.8	132.4±81.3	99.7±34.5	115.9±83.3	0.699
Creatinine	64.8±17.3	57.8±22.8	61.7±28.0	49.4±10.8	0.372
Urea	3.1±0.5	3.9±2.0	4.1±2.1	2.9±0.6	0.203
FVC%	87.6 ±15.6	61.4±11.4	57.3±11.6	94.1±16.4	<0.001
FEV1%	61.8 ±12.3	56.9±11.2	43.0±9.6	88.1±18.4	<0.001
Freq of crisis/year	0.5±0.8	1.5±1.3	1.1±0.9	1.1±1.4	0.410
TRV(m/s)	2.4±0.3	2.3±0.4	2.6±0.4	2.2±0.4	0.056

**Table 7:** Association between FEV1, FVC percentage predicted, and TRV values.

	TRV		X^2^-value
	Normal n(%)	Elevated n(%)	
*FEV1 *%* Predicted*			
< 80%	36 (67.9)	17(32.1)	0.570
> 80%	12(66.7)	6(33.3)	

*FVC *%* predicted*			
< 80%	29(65.9)	15(34.1)	0.452
> 80%	19(70.4)	8(29.6)	

## Data Availability

The data used to support the findings of this study are available from the corresponding author upon request.
